# Supplementary material for: MARTS-DB: a database of mechanisms and reactions of terpene synthases
Source: BMC Bioinformatics. 2025 Dec 8;27:10. doi: 10.1186/s12859-025-06341-8 (PMC12797696; doi:10.1186/s12859-025-06341-8)
Supplement: Supplementary file 1 — Supplementary Material 1 [file 12859_2025_6341_MOESM1_ESM.docx]

# Supplementary information

| **Step type** | **Description** |
| --- | --- |
| Dephosphorylation | Abstraction of the diphosphate group, the initiation step for class I TPS reactions [[1, 2]](https://paperpile.com/c/HBZCx9/G7qc+BCgs). |
| Protonation-class II | Protonation of an alkene or epoxide bond, initiating a class II TPS reaction [[9]](https://paperpile.com/c/HBZCx9/nOYl). |
| Cyclization | Nucleophilic attack of a double bond on the positive charge of a carbocation, forming a new cycle within the molecule [[2]](https://paperpile.com/c/HBZCx9/BCgs). |
| Hydride shift | Transfer of a single hydride ion between a carbon atom and a carbocation [[51]](https://paperpile.com/c/HBZCx9/KQlU). |
| Methyl shift | Transfer of a methyl group between a carbon atom and a carbocation [[1, 52, 53]](https://paperpile.com/c/HBZCx9/0Suv+Eiiy+G7qc). |
| WM rearrangement | Wagner–Meerwein rearrangement, typically a 1,2 migration of a ring carbon atom in a polycyclic system. In TPS literature and MARTS-DB, the term applies to any shift in the ring skeleton [[54, 55]](https://paperpile.com/c/HBZCx9/Gf9L+eYz7). |
| Proton transfer | Transfer of a proton between a hydrogen atom and a double bond, resulting in a repositioning of a double bond on the positive charge [[56, 57]](https://paperpile.com/c/HBZCx9/3PPM+Ilrl). |
| Bond cleavage | Cleavage of a C–C bond, resulting in the formation of a new double bond [[58–60]](https://paperpile.com/c/HBZCx9/y9sW+nlU6+78kw). |
| Oxygen cyclization | Nucleophilic attack reaction of an OH group on a carbocation, abstracting the alcohol hydrogen and forming a heterocycle [[61, 62]](https://paperpile.com/c/HBZCx9/ZOYj+5RjJ). |
| Phosphorylation | Re-addition of the abstracted pyrophosphate to the carbocation. Via phosphorylation, TPSs achieve isomerization of a *trans*-allylic cation to a *cis*-allylic cation, the first step in monoterpene and some sesquiterpene and diterpene biosynthesis [[1, 63, 64]](https://paperpile.com/c/HBZCx9/G7qc+zVFv+LONb). |
| Protonation | Protonation of a stable intermediate formed during the reaction cascade [[65–67]](https://paperpile.com/c/HBZCx9/h7Vc+TSBQ+nEiz). |
| Deprotonation | Abstraction of a proton from the carbocation, forming a double bond [[65, 68]](https://paperpile.com/c/HBZCx9/J3uv+h7Vc). |
| Hydroxylation | Nucleophilic attack of water on a carbocation, forming an alcohol and ending the reaction cascade [[18, 69]](https://paperpile.com/c/HBZCx9/uY93+HMEf). |
| Cyclopropanation deprotonation | Rare termination of the reaction cascade where cyclization coupled with deprotonation forms a cyclopropyl ring [[66, 70]](https://paperpile.com/c/HBZCx9/AT7M+TSBQ). |
| Fragmentation | Observed in terpenes created from unusual precursors with non-canonical carbon numbers (for example, C_16_), where the carbocation fragments into two parts [[52, 71]](https://paperpile.com/c/HBZCx9/0Suv+PYRt). |
| Cycloaddition | Mending of a fragmented carbocation by cycloaddition [[52]](https://paperpile.com/c/HBZCx9/0Suv). |

**Table S1**. The 16 mechanism steps used in MARTS-DB to describe the reaction mechanisms of terpene synthases.
